# Type 2 diabetes mellitus aggravates coronary atherosclerosis in hypertensive individuals based on coronary CT angiography: a retrospective propensity score-based study

**DOI:** 10.3389/fcvm.2024.1372519

**Published:** 2024-05-20

**Authors:** Yu Jiang, Zhi-gang Yang, Jin Wang, Li Jiang, Pei-lun Han, Rui Shi, Yuan Li

**Affiliations:** ^1^ Department of Radiology, West China Hospital, Sichuan University, Chengdu, Sichuan, China; ^2^ West China Biomedical Big Data Centre, West China Hospital, Sichuan University, Chengdu, China

**Keywords:** type 2 diabetes mellitus, hypertension, coronary computed tomography angiography, coronary atherosclerosis, cardiovascular disease

## Abstract

**Background:**

The effect of type 2 diabetes mellitus (T2DM) on coronary atherosclerosis detected on coronary computed tomography angiography (CCTA) in hypertensive patients has attracted increasing attention. This study investigated the relationships of T2DM with coronary artery plaque characteristics and semiquantitative CCTA scores in hypertensive patients.

**Materials and methods:**

In this single-center study, 1,700 hypertensive patients, including 850 T2DM [HT(T2DM+)] and 850 non-T2DM [HT(T2DM−)] individuals, were retrospectively analyzed after propensity matching. Plaque type, extent, coronary stenosis, segment involvement score (SIS), segment stenosis score (SSS), and CT-based Leaman score (CT-LeSc) based on CCTA were assessed and compared between the two groups.

**Results:**

HT(T2DM+) patients had more coronary segments with calcified plaque (2.08 ± 2.20 vs. 1.40 ± 1.91), mixed plaque (2.90 ± 2.87 vs. 2.50 ± 2.66), nonobstructive stenosis (4.23 ± 2.44 vs. 3.62 ± 2.42), and obstructive stenosis (1.22 ± 2.18 vs. 0.78 ± 1.51), a lower proportion of 1-vessel disease (15.3% vs. 25.5%), a higher proportion of 3-vessel disease (59.6% vs. 46.7%), and higher SIS (5.5 ± 3.1 vs. 4.4 ± 3.0), SSS (10.3 ± 8.5 vs. 7.7 ± 7.1), and CT-LeSc (9.4 ± 5.6 vs. 7.9 ± 5.2) than HT(T2DM−) patients (all *P*-values <0.05). Multivariable analysis revealed that T2DM was an independent risk factor for calcified plaque [odds ratio (OR) = 2.213], obstructive coronary artery disease (CAD) (OR = 1.271), multivessel disease (OR = 1.838), SIS > 4 (OR = 1.910), SSS > 6 (OR = 1.718), and CT-LeSc > 5 (OR = 1.584) in hypertension population (all *P*-values <0.05).

**Conclusion:**

T2DM was independently associated with the presence of calcified coronary artery plaque and increased the risk of obstructive CAD, multivessel disease, and CT-LeSc > 5 in hypertensive patients. More attention should be given to the assessment and management for coronary atherosclerosis in hypertensive patients with T2DM, as this population may have a higher risk of cardiovascular events.

## 1 Introduction

More than 1 billion people worldwide have been diagnosed with hypertension, and the prevalence and burden of hypertension is expected to continue rising due to population aging (1). Diabetes is also a common chronic disease threatening public health, and it has been estimated that more than 536 million people have been diagnosed with diabetes according to the International Diabetes Federation Diabetes Atlas data (2). Given their shared underlying risk factors and the vicious feedback cycle between them, hypertension and diabetes are closely related to each other and frequently coexist (3–6). Specifically, patients with hypertension have higher concentrations of fasting serum glucose and a higher risk of developing diabetes than patients with normal blood pressure, and the development of type 2 diabetes mellitus (T2DM) is approximately two times as common in patients with hypertension than in normotensive patients (7).

Although the rising trend of hypertension and diabetes is alarming, the rates of awareness and management remain low (8). It is well known that both hypertension and diabetes are leading risk factors for cardiovascular disease, and the coexistence of diabetes with hypertension increases the risk of cardiovascular events (9–11). It has been reported that the risk of cardiovascular disease in patients with both arterial hypertension and diabetes mellitus is at least five times higher than that in individuals without either of the diseases (12). Accurate assessment of coronary atherosclerosis would be helpful to improve risk stratification and is critical for the clinical management of hypertensive patients accompanied with T2DM.

A previous study revealed the feasibility of treatment decision-making based solely on coronary computed tomography angiography (CCTA) in patients with coronary artery disease (CAD) (13). Recently, semiquantitative CCTA-based risk scores have shown great potential for cardiovascular risk prediction and stratification of patients with diabetes mellitus and have found significant differences between patients with and without diabetes mellitus (14–16). However, it remains uncertain whether semiquantitative CCTA-based scores apply well to a specific cohort of hypertensive patients, and the effect of T2DM on coronary atherosclerosis in patients with hypertension is not completely clear. To this end, this study compared multiple semiquantitative CCTA-based scores between hypertensive patients with and without T2DM.

## 2 Materials and methods

### 2.1 Study population

Clinically diagnosed essential hypertensive patients with coronary artery plaques detected by CCTA examination in our hospital from January 2018 to December 2021 were retrospectively included. Hypertension was defined as a documented history of hypertension, treatment with anti-hypertensive medication, or blood pressure ≥140/90 mmHg (17). The CCTAs were performed for preoperative evaluation or screening coronary atherosclerosis in patients at high risk of CAD. The exclusion criteria were as follows: (1) incomplete clinical data; (2) patients underwent coronary artery stenting or bypass grafting before CCTA examination; (3) patients with type 1 diabetes mellitus or prediabetes; or (4) CCTA image quality that was too poor to assess the coronary artery plaque. Hypertensive patients were divided into two groups according to whether they were comorbid with T2DM: patients with [HT(T2DM+)] and without [HT(T2DM−)] T2DM. T2DM was defined according to the criteria of the American Diabetes Association (18). T2DM and non-T2DM patients were subsequently matched based on age, sex, body mass index (BMI), smoking status, hypertension history, systolic blood pressure (SBP), and diastolic blood pressure (DBP) with a 1:1 propensity score matching method. Consequently, 850 T2DM and 850 non-T2DM hypertensive patients were enrolled in this study. This study was approved by the Biomedical Research Ethics Committee of our hospital, and written informed consent was waived.

### 2.2 CCTA scanning protocols

The CCTA scans were performed in the supine position with a Siemens 64-Slice CT, Siemens 128-Slice CT, or GE 256-Slice CT (SOMATOM Definition, Siemens Medical Solutions, Forchheim, Germany; SOMATOM Definition FLASH, Siemens Medical Solutions, Forchheim, Germany; or Revolution CT, GE Healthcare, Waukesha, WI, USA). The CCTA image was acquired by using either a retrospective or prospective electrocardiogram-gated protocol. A beta-blocker was not routinely used for scanning. A nonionized contrast agent was used for intravenous bolus injection. The scan range was from the tracheal bifurcation to 20 mm below the inferior cardiac apex. The reconstruction image layer thickness was set to 0.625 mm.

### 2.3 Image analysis

**Figure 1:**
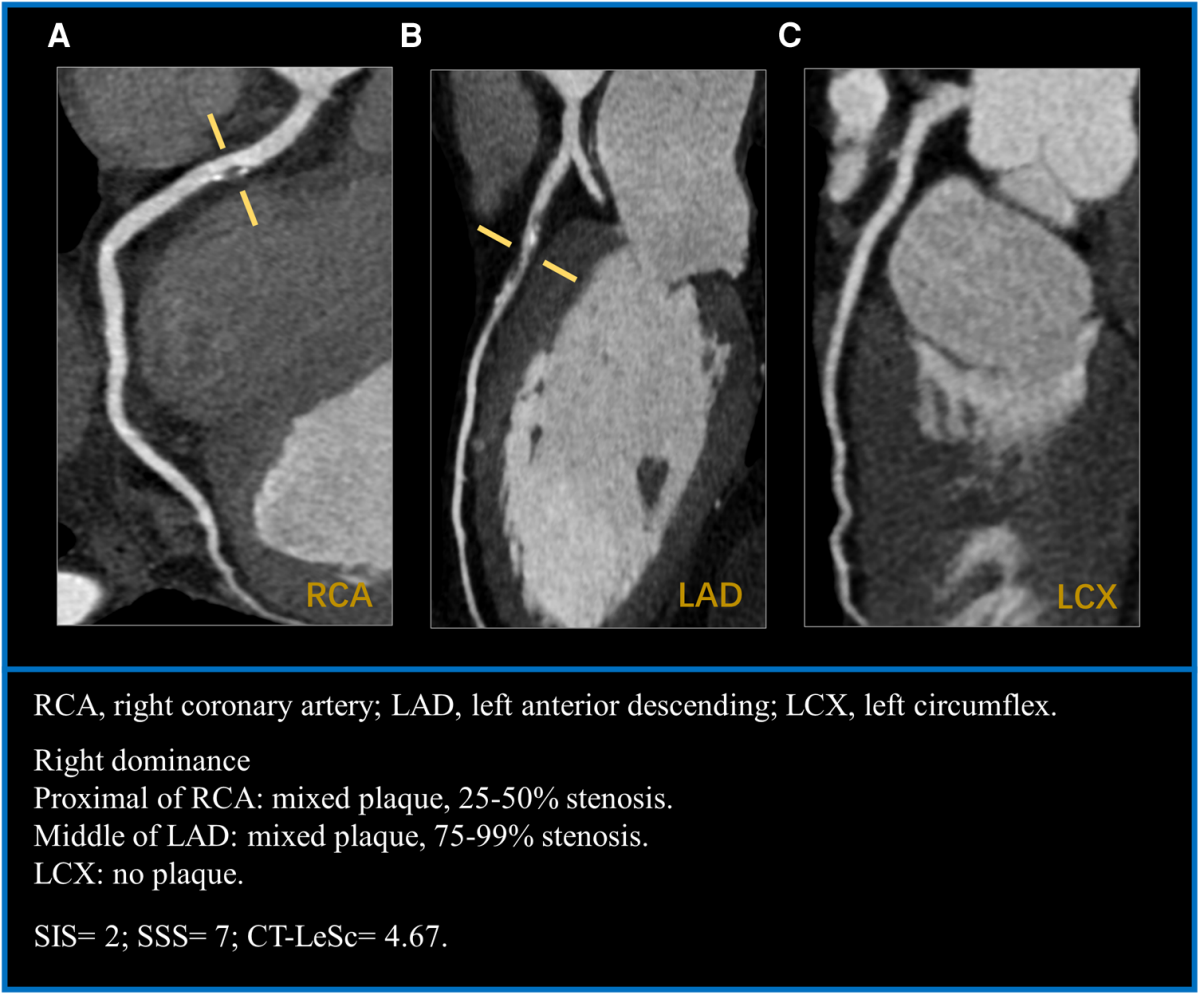
A case example of a 57-years-old man with hypertension for 20 years. (**A**) mixed plaque with mild stenosis in the proximal segment of RCA. (**B**) mixed plaque with severe stenosis in the middle segment of LAD. (**C**) LCX without plaque. CT-LeSc = RCA (1 × 1.5 × 0.615) + LAD (2.5 × 1.5 × 1) = 4.67. RCA, right coronary artery; LAD, left anterior descending; LCX, left circumflex; CT-LeSc, CT-adapted leamanscore.

CCTA images evaluation was performed by two professional radiologists who were blinded to the clinical information of the patients. If there was any disagreement on the image evaluation, they reached a consensus through discussion. For all patients, the type of plaque and lumen stenosis of each coronary segment were evaluated, and the segment involvement score (SIS), segment stenosis score (SSS) and CT-LeSc were calculated. Plaque type was categorized as calcified plaque, noncalcified plaque, and mixed plaque. Stenosis severity was categorized as follows: without stenosis, <25% stenosis, 25%–49% stenosis, 50%–69% stenosis, 70%–99% stenosis, and total occlusion. Nonobstructive stenosis was defined as <50% stenosis, and obstructive stenosis was defined as ≥50% stenosis. Any finding of obstructive stenosis was defined as obstructive CAD. More than one coronary vessel detected with plaque was considered multivessel disease. SIS was calculated as the sum of coronary artery segments with plaque (range 0–17). SSS was calculated as the sum of the grade of stenosis severity (0–5) of each segment (range 0–85). The evaluation of the coronary segment in each patient was based on a modified 17-segment model according to the American Heart Association (19). CT-LeSc was calculated for each patient according to weighting factors including plaque composition (noncalcified/mixed or calcified), plaque location, and lumen stenosis (<50% or ≥50% stenosis) (Supplementary File Figure S1) (20). Figure 1 shows an example for analysis of CCTA semiquantitative scores.

### 2.4 Statistical analysis

Categorical data are presented as numbers and percentages, and continuous variables are reported as the mean ± standard deviation. Coronary plaque type, extent of plaque, lumen stenosis, SSS and CT-LeSc were compared between HT(T2DM+) and HT(T2DM−) patients. The nonparametric Mann–Whitney test was used to evaluate differences in continuous variables, and the *χ*^2^ test was used to compare categorical variables. Univariable and multivariable analyses were used to analyze the associations of T2DM with CCTA findings and semiquantitative CCTA score in patients with hypertension, and the univariable and multivariable odds ratios (OR) with 95% confidence intervals (CIs) were obtained using logistic regression analysis. Differences were regarded as significant when they were associated with a two-tailed *P* < 0.05. SPSS version 25.0 was used for statistical analyses.

## 3 Results

### 3.1 Study population

The baseline characteristics of the hypertensive patients are listed in Table 1. Overall, 1,700 individuals, including 850 HT(T2DM+) and 850 HT(T2DM−) patients, with a mean age of 70.2 ± 9.7 years, of whom 615 (36.2%) were female were eventually analyzed. HT(T2DM+) patients were largely comparable to HT(T2DM−) patients regarding sex, age, BMI, smoking history, duration of hypertension, SBP and DBP (all *P*-values >0.05). However, compared with the HT(T2DM−) group, the HT(T2DM+) group had higher levels of fasting blood glucose and triglyceride, lower levels of cholesterol, high-density lipoprotein cholesterol and low-density lipoprotein cholesterol and more patients receiving ACEI/ARB, statin and antiplatelet therapy (all *P*-values <0.05).

**Table 1:** Comparison of baseline characteristics between hypertension patients with and without T2DM.

	HT(T2DM−) (*n* = 850)	HT(T2DM+) (*n* = 850)	*P*-value
Female, *n* (%)	308 (36.2%)	307 (36.1%)	0.960
Age, years	70.2 ± 9.7	70.2 ± 9.7	0.936
BMI, kg/m^2^	24.5 ± 3.2	24.6 ± 3.2	0.619
Smoker, *n* (%)	283 (33.3%)	304 (35.8%)	0.284
Duration of hypertension, years	11.6 ± 10.1	11.9 ± 9.3	0.135
SBP, mmHg	140.1 ± 19.3	140.5 ± 19.2	0.824
DBP, mmHg	81.4 ± 13.0	81.3 ± 12.8	0.382
Duration of diabetes, years	–	9.4 ± 7.6	–
Laboratory data			
HbA1c (%)	–	7.5 ± 1.5	–
Fasting blood glucose, mmol/L	5.10 ± 0.57	7.69 ± 2.66	<0.001
Cholesterol, mmol/L	4.10 ± 1.09	3.99 ± 1.14	0.005
Triglyceride, mmol/L	1.43 ± 0.81	1.62 ± 1.16	<0.001
HDL-C, mmol/L	1.21 ± 0.36	1.13 ± 0.31	<0.001
LDL-C, mmol/L	2.35 ± 0.91	2.25 ± 0.93	0.006
eGFR, ml/min/1.73 m^2^	77.9 ± 17.1	79.0 ± 18.1	0.261
Medications, *n* (%)			
ACEI/ARB	285 (33.5%)	334 (39.3%)	0.014
Beta-blocker	165 (19.4%)	136 (16.0%)	0.065
Calcium-channel blocker	497 (58.5%)	464 (54.6%)	0.106
Statins	178 (20.9%)	218 (25.6%)	0.022
Antiplatelet	213 (25.1%)	267 (31.4%)	0.004
Oral medication for T2DM	–	621 (73.1%)	–
Insulin	–	262 (30.8%)	–

Data are presented as mean ± standard deviation or numbers (percentage).

T2DM, type 2 diabetes mellitus; HT, hypertension; BMI, body mass index; SBP, systolic blood pressure; DBP, diastolic blood pressure; HDL-C, high-density lipoprotein cholesterol; LDL-C, low-density lipoprotein cholesterol; eGFR, estimated glomerular filtration rate; ACEI, angiotensin converting enzyme inhibitor; ARB, angiotensin II receptor blocker.

### 3.2 Comparison of conventional CCTA characteristics and semiquantitative CCTA risk scores

The CCTA findings and semiquantitative scores of HT(T2DM+) and HT(T2DM−) patients are presented in Tables 2, 3.

**Table 2:** CCTA findings of hypertension patients stratified by T2DM.

	HT(T2DM−) (*n* = 850)	HT(T2DM+) (*n* = 850)	*P*-value
Plaque type			
Calcified plaques	1.40 ± 1.91	2.08 ± 2.20	<0.001
Mixed plaques	2.50 ± 2.66	2.90 ± 2.87	0.003
Noncalcified plaques	0.51 ± 0.98	0.46 ± 0.88	0.538
Lumen stenosis			
Nonobstructive stenosis	3.62 ± 2.42	4.23 ± 2.44	<0.001
Obstructive stenosis	0.78 ± 1.51	1.22 ± 2.18	0.001
Diseased vessels			
1-vessel	217 (25.5%)	130 (15.3%)	<0.001
2-vessel	236 (27.8%)	213 (25.1%)	0.206
≥3-vessel	397 (46.7%)	507 (59.6%)	<0.001
Obstructive CAD	287 (33.8%)	338 (39.8%)	0.010

Data are presented as mean ± standard deviation or numbers (percentage).

CCTA, coronary computed tomography angiography; T2DM, type 2 diabetes mellitus; HT, hypertension; CAD, coronary artery disease.

**Table 3:** Semiquantitative CCTA risk scores in hypertension patients stratified by T2DM status.

	HT(T2DM−) (*n* = 850)	HT(T2DM+) (*n* = 850)	*P*-value
SIS	4.4 ± 3.0	5.5 ± 3.1	<0.001
SSS	7.7 ± 7.1	10.3 ± 8.5	<0.001
CT-LeSc	7.9 ± 5.2	9.4 ± 5.6	<0.001
CT-LeSc category			<0.001
CT-LeSc ≤ 5	298 (35.1%)	214 (25.2%)	
CT-LeSc > 5	552 (64.9%)	636 (74.8%)	

Data are presented as mean ± standard deviation or numbers (percentage).

CCTA, coronary computed tomography angiography; T2DM, type 2 diabetes mellitus; HT, hypertension; SIS, segment involvement score; SSS, segment stenosis score; CT-LeSc, CT-adapted Leaman score.

**Figure 2:**
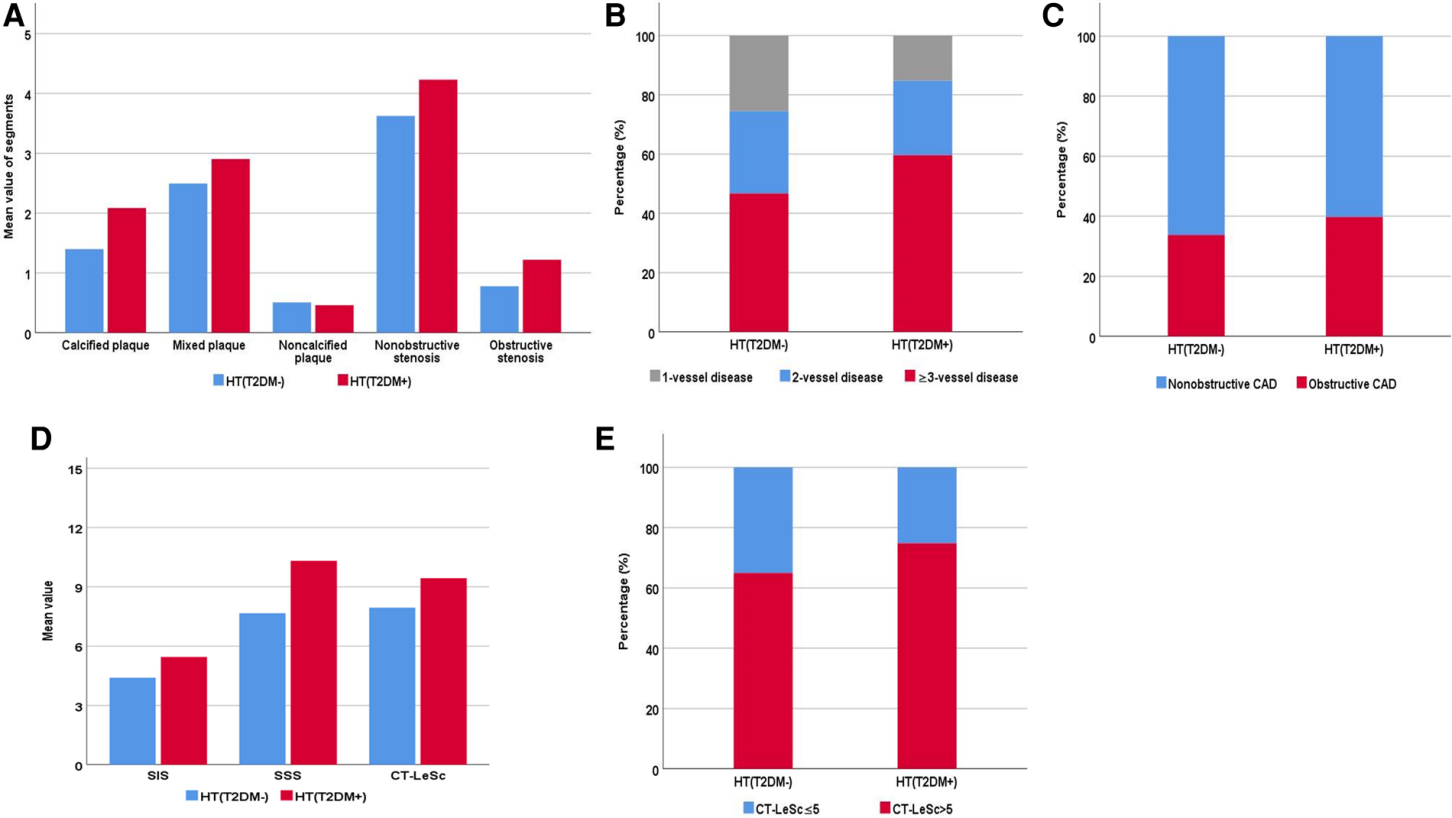
Coronary computed tomography angiography characteristics and semiquantitative score of hypertensive patients with and without type 2 diabetes mellitus [HT(T2DM+) and HT (T2DM−), respectively]. (**A**) The number (mean value) of coronary artery segments of different types of plaque with obstructive and nonobstructive stenosis. (**B**) The proportion of obstructive and nonobstructive coronary artery disease (CAD). (**C**) The proportion of 1-vessel, 2-vessel, and ≥3-vessel disease. (**D**) The mean value of the segment involvement score (SIS), segment stenosis score (SSS), and CT-adapted leaman score (CT-LeSc). (**E**) The proportion of CT-LeSc ≤ 5 and CT-LeSc > 5 disease.

Patients in the HT(T2DM+) group had more coronary segments with calcified plaque (2.08 ± 2.20 vs. 1.40 ± 1.91, respectively), mixed plaque (2.90 ± 2.87 vs. 2.50 ± 2.66, respectively), nonobstructive stenosis (4.23 ± 2.44 vs. 3.62 ± 2.42, respectively), and obstructive stenosis (1.22 ± 2.18 vs. 0.78 ± 1.51, respectively) than HT(T2DM−) patients (all *P*-values <0.05) (Figure 2A). In addition, the HT(T2DM+) group had a higher proportion of patients with obstructive CAD than the HT(T2DM−) group (39.8% vs. 33.8%, respectively; *P* < 0.05) (Figure 2B). Regarding diseased vessels, the HT(T2DM+) group had a lower proportion of patients with 1-vessel disease (15.3% vs. 25.5%, respectively) but a higher proportion of patients with 3-vessel disease (59.6% vs. 46.7%, respectively) than the HT(T2DM−) group (both *P*-values <0.05) (Figure 2C).

As shown in Table 3, patients in the HT(T2DM+) group had higher SIS (5.5 ± 3.1 vs. 4.4 ± 3.0, respectively), SSS (10.3 ± 8.5 vs. 7.7 ± 7.1, respectively), and CT-LeSc (9.4 ± 5.6 vs. 7.9 ± 5.2, respectively) than patients in the HT(T2DM−) group (all *P*-values <0.05) (Figure 2D). In addition, the CT-LeSc > 5 (74.8% vs. 64.9%, respectively) was significantly more prevalent in the HT(T2DM+) group than in the HT(T2DM–) group (*P*-value <0.05) (Figure 2E).

### 3.3 Univariable and multivariable regression analyses of CCTA findings and semiquantitative CCTA risk scores

**Figure 3:**
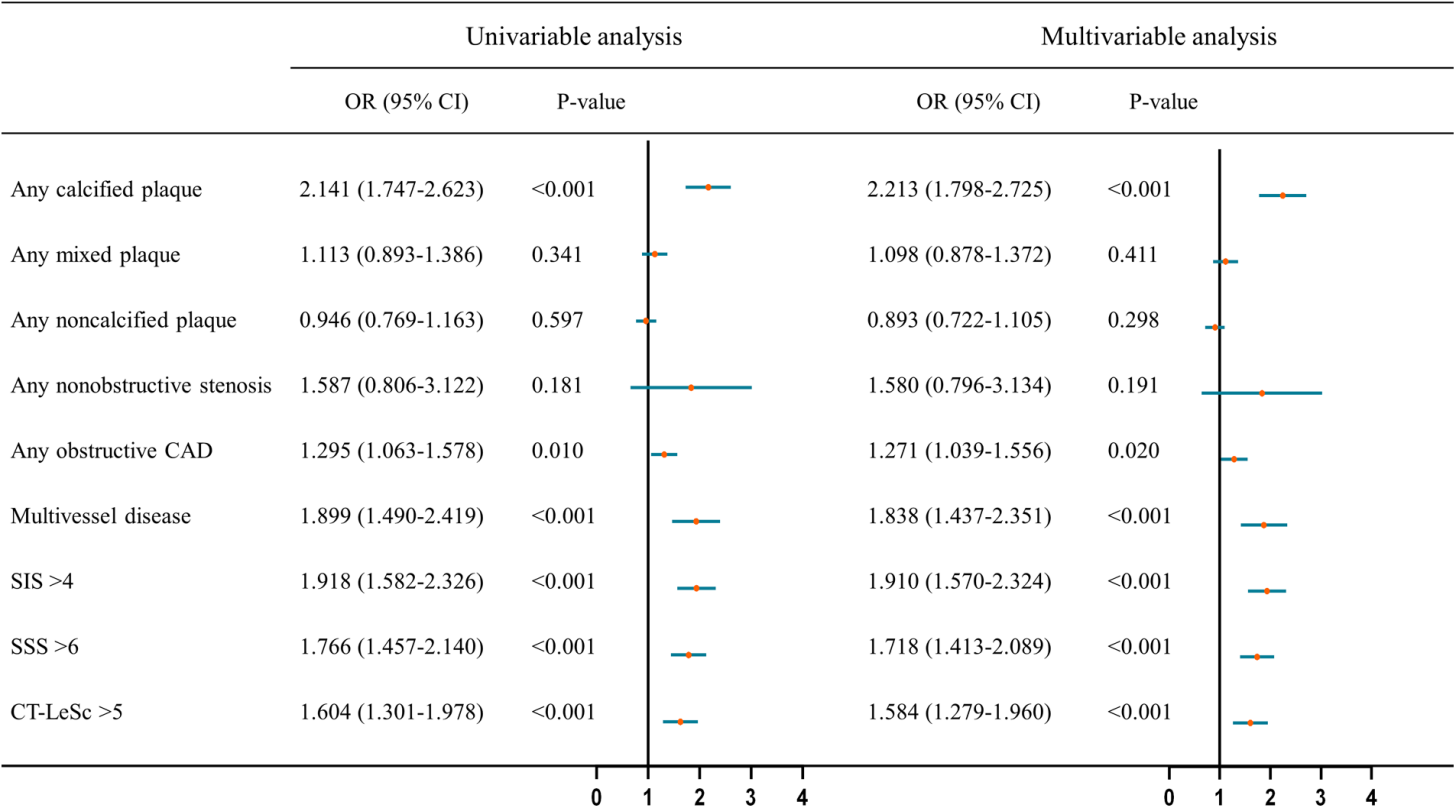
Univariable and multivariable analysis of association of CCTA findings and semiquantitative CCTA score with T2DM in hypertensive patients. The multivariable analysis was adjusted for cholesterol, triglyceride, high-density lipoprotein cholesterol, low-density lipoprotein cholesterol, ACEI/ARB, statins and antiplatelet therapy. CCTA, coronary computed tomography angiography; T2DM, type 2 diabetes mellitus; HT, hypertension; OR, odds ratio; CI, confidence interval; CAD, coronary artery disease; SIS, segment involvement score; SSS, segment stenosis score; CT-LeSc, CT-adapted leaman score.

In the univariable analysis, T2DM was associated with the presence of calcified plaque, obstructive CAD, multivessel disease, SIS > 4, SSS > 6, and CT-LeSc > 5 (all *P*-values <0.05) in hypertensive patients (Figure 3). By multivariable analysis, when adjusting for cholesterol, triglycerides, high-density lipoprotein cholesterol, low-density lipoprotein cholesterol, ACEI/ARB, statin and antiplatelet therapy, T2DM was found to be an independent risk factor for the presence of calcified plaque [OR (95% CI): 2.213 (1.798–2.725)], obstructive CAD [OR (95% CI): 1.271 (1.039–1.556)], multivessel disease [OR (95% CI): 1.838 (1.437–2.351)], SIS > 4 [OR (95% CI): 1.910 (1.570–2.324)], SSS > 6 [OR (95% CI): 1.718 (1.413–2.089)], and CT-LeSc > 5 [OR (95% CI): 1.584 (1.279–1.960)] (all *P*-values <0.05) in the hypertension population (Figure 3).

## 4 Discussion

This study explored the impact of T2DM on coronary atherosclerosis in hypertensive patients by comparing CCTA findings and semiquantitative CCTA scores between hypertensive individuals with and without T2DM. After adjusting for confounding factors, T2DM status was associated with the presence of calcified coronary artery plaque in hypertensive patients. T2DM was related to the increased risk of obstructive CAD in hypertensive patients. In addition, T2DM increased the risks of multivessel disease, higher SIS and SSS values, and disease of CT-LeSc > 5 in patients with hypertension.

Hypertension is a common comorbidity among diabetes patients, and hypertensive patients exhibit a higher risk of diabetes than normotensive patients (6, 21). Compared with normotensive patients, hypertensive patients are more likely to develop coronary atherosclerosis and suffer from cardiovascular events (22). The presence of diabetes promotes endothelial activation and macrophage polarization toward an inflammatory phenotype, thus promoting the development of atherosclerotic plaques (23). We could infer that the presence of diabetes mellitus in hypertensive populations could progress the severity of coronary atherosclerosis. However, the specific difference of characteristics of coronary atherosclerosis in hypertensive individuals with and without diabetes should be further explored. Thus, in this study, we focused on the coronary atherosclerosis characteristics based on CCTA findings from hypertensive population with and without diabetes.

Although current diagnostic modalities for CAD mainly focus on ischemic lesions, plaque composition and morphology are developing as strong predictors for cardiac events even without confirmed ischemia (24). As shown in previous data, a higher burden of calcification in patients with stable CAD predicts long-term cardiovascular event occurrence (25). Accumulating evidence has revealed that vascular calcification is a complex biological process involving diverse pathophysiological mechanisms in which the phosphorous and calcium milieu and vascular smooth muscle cells play important roles, and vascular calcification is considered a vascular pathological disorder related to a variety of diseases (23, 26). Regardless of the mechanism for arterial endothelial damage, both hypertension and diabetes mellitus may result in arterial calcification and could be predictors for the presence of coronary artery calcification (27, 28). There is an important thing in the management of coronary atherosclerosis is that calcified plaque is rarely modifiable (29). Thus, the result of our study that T2DM was independently associated with the presence of calcified plaques in hypertensive patients shows importance of calcification screening and indicates a requirement for an additional therapy other than strategy of plaque regression alone in such population.

The present study demonstrated that T2DM was independently associated with obstructive CAD but not with the presence of nonobstructive stenosis in hypertensive patients. This may be explained by the high endothelial shear stress exist in patients with hypertension or diabetes. Hypertension results in a persistent elevation in the vessel wall shear stress, and arteries experience increased stiffness and diminished compliance with prolonged exposure to elevated blood pressure (30). Patients with diabetes mellitus exhibit heightened red blood cell aggregation and blood viscosity, thereby potentially amplifying shear stress (30). High endothelial shear stress has the potential to induce endothelial damage, facilitate platelet deposition and potentially contribute to the development of constrictive vascular remodeling characterized by fibroproliferation and luminal stenosis (31). Thus, the presence of diabetes in hypertensive patients are more likely to promote to the lumen narrowing and lead to obstructive CAD.

Traditionally, elevated blood pressure increases the pulsatile wall stress, thereby promoting elastin degradation and leading to long-term atherosclerosis; however, the opposite may be true that arterial stiffness may contribute to the progress of hypertension (3). It is known that hyperglycemia in patients with diabetes mellitus damages the cardiovascular system and is associated with low-grade inflammation and accelerated atherosclerosis (32, 33). From this, the presence of T2DM may aggravate the adverse effect of hypertension and coronary atherosclerosis, which may explain the fact that T2DM was independently associated with the presence of multivessel disease in hypertensive patients in the present study. Patients with hypertension and T2DM have higher all-cause and cardiovascular mortality than patients with hypertension or T2DM alone (34). In addition to hypertension, the additional risk of T2DM may be related to the amplification of vascular injury (3, 4). The evaluation of semiquantitative CCTA scores has great ability in risk stratification and could be more useful for nonfatal myocardial infarction or all-cause death discrimination than binary assessment of obstructive stenosis alone (15). Coronary atherosclerosis of CT-LeSc > 5 has been reported to show a greater risk of cardiovascular events (35). Our study also found that T2DM was independently associated with the presence of disease with CT-LeSc > 5 in hypertensive patients, which may indicate a higher risk of cardiovascular events in this population. Therefore, the assessment of semiquantitative CCTA scores is important for clinical management in hypertensive patients with T2DM.

The current study has some limitations. First, this was an observational cross-sectional study, and the value of serial CCTA to explore the natural process of atherosclerosis in hypertensive patients with T2DM remains unclear. Second, this retrospective study did not include follow-up information. In the future, further verification of the relationship of semiquantitative CCTA scores with cardiovascular events in hypertensive patients with T2DM is needed. Third, coronary artery plaque analysis was based on visual categorization, qualitative or semiquantitative assessment in this study. Further quantitative research needs to be established to obtain additional useful information.

## 5 Conclusion

T2DM was independently associated with the presence of calcified coronary artery plaque and increased the risk of obstructive CAD, multivessel disease, and CT-LeSc > 5 in hypertensive patients. More attention should be given to the assessment and management for coronary atherosclerosis in hypertensive patients with T2DM, as the results may indicate a higher risk of cardiovascular events in this population.

## Data Availability

The raw data supporting the conclusions of this article will be made available by the authors, without undue reservation.
