# Supplementary material for: Type 2 diabetes mellitus aggravates coronary atherosclerosis in hypertensive individuals based on coronary CT angiography: a retrospective propensity score-based study
Source: Front Cardiovasc Med. 2024 May 20;11:1372519. doi: 10.3389/fcvm.2024.1372519 (PMC11149417; doi:10.3389/fcvm.2024.1372519)
Supplement: Supplementary file 1 [file Datasheet1.docx]

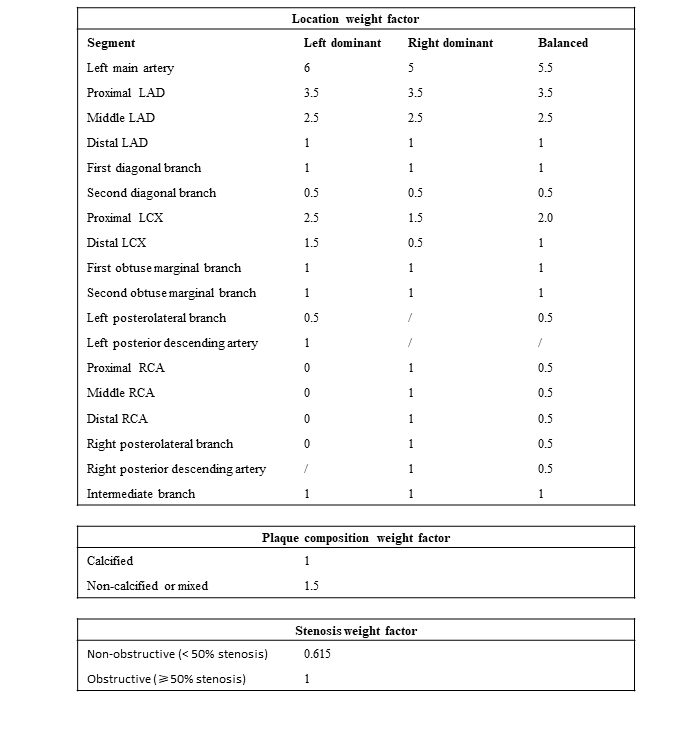


**Figure S1** **CT-based Leaman score (CT-LeSc) calculation.** CT-LeSc is calculated by the summation of multiplication of the location weight factor, the plaque composition weight factor, and the stenosis weight factor of each coronary artery segment. LAD, left anterior descending artery; LCX, left circumflex artery; RCA, right coronary artery.
